# Incidence of adverse perinatal outcomes in highly vulnerable pregnant women – the Mothers of Rotterdam study

**DOI:** 10.1186/s12884-025-07401-w

**Published:** 2025-04-11

**Authors:** Kajal S. C. Mohabier, Hanneke P. de Graaf, Eric A. P. Steegers, Loes C. M. Bertens

**Affiliations:** 1 Department of Obstetrics and Gynaecology, Erasmus University Medical Centre Rotterdam, P.O. Box 2040, 3000 CA Rotterdam, The Netherlands; 2 Department of Obstetrics and Gynaecology, Division of Obstetrics and Prenatal Medicine, Erasmus University Medical Centre Rotterdam, Rotterdam, The Netherlands

**Keywords:** Socioeconomic disadvantage, Vulnerable populations, Pregnancy, Adverse pregnancy outcomes, Preterm birth, Small for gestational age

## Abstract

**Background:**

Socioeconomic disadvantaged circumstances are known to affect health outcomes, but during pregnancy it also affects the growth and development of the fetus. This often results in adverse perinatal outcomes and other long lasting effects. Here we refer to pregnant women living in such circumstances as a highly vulnerable population.

**Objectives:**

To study adverse perinatal outcomes in highly vulnerable pregnant women within the Mothers of Rotterdam (MoR) study and to compare findings to the outcomes of women in the Netherlands as a whole and the city of Rotterdam.

**Methods:**

Pregnancy and childbirth data from women participating in the MoR study (2015–2019) was requested from their obstetric professional. For comparison, data from the Dutch national birth registry (Perined) were used representing women in the Netherlands and Rotterdam. Main outcome measures were preterm birth (PTB) and small for gestational age (SGA). Secondary outcome measures were perinatal mortality and a low Apgar score. Only singleton viable pregnancies (i.e. birthweight above 500 g or born after 22 + 0 weeks of gestation) were included in this study.

Prevalence rates and corresponding 95% confidence intervals (95%CI) were calculated for all outcomes in each group. Direct standardization was used to account for possible differences in case-mix composition between the studied groups.

**Results:**

Data on 346 childbirths within the MoR study were retrieved and compared to 813,755 and 34,009 childbirths in the Netherlands and Rotterdam, respectively. The prevalence of PTB (4.34% (95%CI 2.19–6.48) was lower in the MoR population compared to both the Netherlands (6.21% (95%CI 6.16–6.27)) and Rotterdam (6.39% (95%CI 6.13–6.65)). The prevalence of SGA (21.09% (95% CI 16.80–25.40)) was higher in the MoR population compared to both the Netherlands (10.11% (95%CI 10.04–10.17)) and Rotterdam (13.28% (95%CI 12.92–13.65)). There were no cases of perinatal mortality registered in the MoR population. The prevalence of a low Apgar score (0.87% (95%CI 0.00–1.84)) was lower in the MoR population.

**Conclusions:**

Our study found unexpected low PTB and high SGA prevalence rates in the MoR population compared to the Netherlands and Rotterdam. Mechanisms through which socioeconomic disadvantaged circumstances affect perinatal health seem to work differently in various strata of vulnerable populations.

**Supplementary Information:**

The online version contains supplementary material available at 10.1186/s12884-025-07401-w.

## Introduction

Despite growing advances in health care over the past few decades, health inequalities still remain present worldwide and pose a complex challenge in the field of public health. There is a growing body of literature linking a low socioeconomic status (SES) and accompanying disadvantaged circumstances to suboptimal health and well-being of individuals, with lower levels of health literacy and more risky health behaviour being identified as important mechanisms [1–3]. Prolonged exposure to stress as a result of disadvantaged circumstances is believed to be another important mechanism in this association.

The negative effects of the disadvantaged circumstances are already transferred from one generation to another before and during pregnancy, with long lasting effects throughout the entire life course [4–7]. The preconception period and pregnancy are crucial moments for the growth and development of the fetus, with maternal health as an important determinant. Suboptimal maternal health due to disadvantaged circumstances often leads to suboptimal growth and development of the fetus. This can subsequently result in adverse perinatal outcomes, but is also associated with an increased risk of behavioural, developmental and health related problems in early childhood and later in life [4–10]. Considering the disadvantaged circumstances, exposure to stress and transgenerational transmission, we refer to pregnant women living in such circumstances as a highly vulnerable population.

Within Rotterdam, the second largest city of the Netherlands, 12.8% of households have a low income and 14.3% of children in Rotterdam grow up in poverty, which is twice as high as the average in the Netherlands [11]. The municipality of Rotterdam and the Erasmus Medical Centre, along with several other partners, have combined their efforts in the Mothers of Rotterdam (MoR) program [12]. Within the MoR program, (targeted) social care is provided to highly vulnerable pregnant women with the aim to support them and their children.

The aim of this article is to study adverse perinatal outcomes in highly vulnerable pregnant women within the MoR study. To provide context to our findings, the same perinatal outcomes will be described for both the Netherlands as a whole, and the city of Rotterdam. We hypothesized that the prevalence of adverse perinatal outcomes was higher in the MoR population compared to the Netherlands and the city of Rotterdam.

## Methods

### Recruitment

The MoR program provides targeted social care with a holistic approach by integrating medical and social care for highly vulnerable pregnant women and their (unborn) children from pregnancy until the second birthday of the child, while targeting adult and child issues simultaneously [12]. The MoR study is a prospective cohort study designed to evaluate the effectiveness of targeted social care provided within the MoR program compared to standard social care.

Recruitment for the MoR study (January2016—December2020) was open to all pregnant women (irrespective of gestational age) who resided in Rotterdam, were considered to be highly vulnerable, and were referred to, or applied for, social care [12]. Eligibility and care need were assessed in the home environment of the pregnant woman by social care professionals filling out a vulnerability checklist developed explicitly for the care in the MoR study. The checklist consisted of 47 adversities divided over 8 life-domains (see Appendix 1). Pregnant women facing a minimum of three adversities over at least two life-domains on the vulnerability checklist were considered to be highly vulnerable. Both targeted and standard social care provide tailored care for identified adversities. However, with its holistic approach, targeted social care also focuses on the prevention of adversities in the long term.

Women participating in the study gave explicit written informed consent to data collection, including retrieval of pregnancy and childbirth data from involved obstetric care providers. Social care provision to these women was independent of their participation in the MoR study. Ethical approval for the study was obtained from the Erasmus Medical Centre Ethics Committee (MEC-2016–012).

### Data collection

For the MoR population, maternal and social characteristics were retrieved from the application forms as an integral part of the study. Records on pregnancy and childbirth characteristics were retrieved from involved obstetric care providers for all participants who consented for data retrieval.

Records from the Dutch Perinatal Registry (Perined) were used to obtain maternal, social, pregnancy and childbirth characteristics of women in the Netherlands and Rotterdam [13]. Perined contains routine care information from midwives, gynaecologists and paediatricians, covering 98% of all pregnancies and childbirths in the Netherlands. National childbirth outcomes (2015—2019) were available and extracted after approval of the executive board of Perined (PRN 20.10). Data specific for Rotterdam could be extracted from the data in Perined based on postal codes. National data for 2020 was unavailable at the moment of data extraction, thus data from 2015–2019 was used to cover a consistent cohort duration.

### Outcome measures

Main outcome measures were preterm birth (PTB; < 37 weeks of gestation) and small for gestational age (SGA; birthweight < p10 for gestational age, according to the reference curves) [14]. Secondary outcome measures were perinatal mortality (foetal mortality > 22 weeks of gestation and neonatal mortality up to 7 days after birth), a low Apgar score (Apgar score ≤ 7, 5 min after birth). Only singleton viable pregnancies (birthweight above 500 g or born after 22 + 0 weeks of gestation) were included in this study.

### Determinants

The baseline maternal, social and pregnancy characteristics included gestational age at application for social care, maternal age (< 20, 21–25, 26–30, 31–35, and > 35 years), parity (primiparous and multiparous), living in a deprived neighbourhood, ethnicity (western (i.e. Caucasian/European), non-western, and unknown) and marital status (married, divorced, single, other, unknown).

The postal code of a participant at the start of social care was used to determine if they were living in a deprived neighbourhood according to the guidelines of the Dutch Healthcare Authority (NZa) [15]. The deprivation index is based on the proportion of non-active persons (i.e., unemployed or not working individuals), mean individual income, mean address density and the proportion of non-western immigrants per neighbourhood[16]. Ethnicity was extracted from the obstetric files and registered in PRN according to the discretion of the woman’s obstetric healthcare professional. Marital status of the population of women in the Netherlands, with a similar age-range as the MoR population, was retrieved from the StatLine electronic database (Statistics Netherlands) [17].

Childbirth characteristics included information on location of childbirth (at home, hospital, birthing centre or unknown), method of childbirth (spontaneous vaginal delivery, vaginal delivery after induction, scheduled caesarean section, emergency caesarean section or vacuum assisted delivery), postpartum haemorrhage (defined as blood loss > 1000 ml within the first 24 h following childbirth), congenital anomalies at birth, and perinatal outcomes (both main and secondary outcomes).

### Expected prevalence rates

To account for possible differences in case-mix composition between the groups (i.e. MoR vs. the Netherlands and Rotterdam), expected prevalence rates were calculated with the direct standardization method [18]. With this method the case-mix structure of one population (here the Netherlands as a whole) is used as reference and used to take differences in this case-mix between populations into account. Case-mix variables considered in the current study were maternal age (< 20, 21–30, and > 30 years), parity and ethnicity. The reference population is stratified into all different combinations of the selected case-mix variables, and prevalence rates per stratum are calculated. The other populations are stratified in the same way, to calculate their prevalence rates per stratum. These rates are then corrected for the prevalence rates of the corresponding subgroups in the reference population. Lastly, the prevalence rates of each stratum is combined into a single, expected (standardized) prevalence rate.

### Statistical analyses

Baseline maternal, social, pregnancy and childbirth characteristics were tabulated for all three groups (i.e. MoR population, the Netherlands and Rotterdam). Crude and expected prevalence rates with corresponding 95% confidence intervals (95%CI) for all perinatal outcomes were calculated for each group. All analyses were performed using SPSS version 28.

## Results

### MoR population

**Fig. 1:**
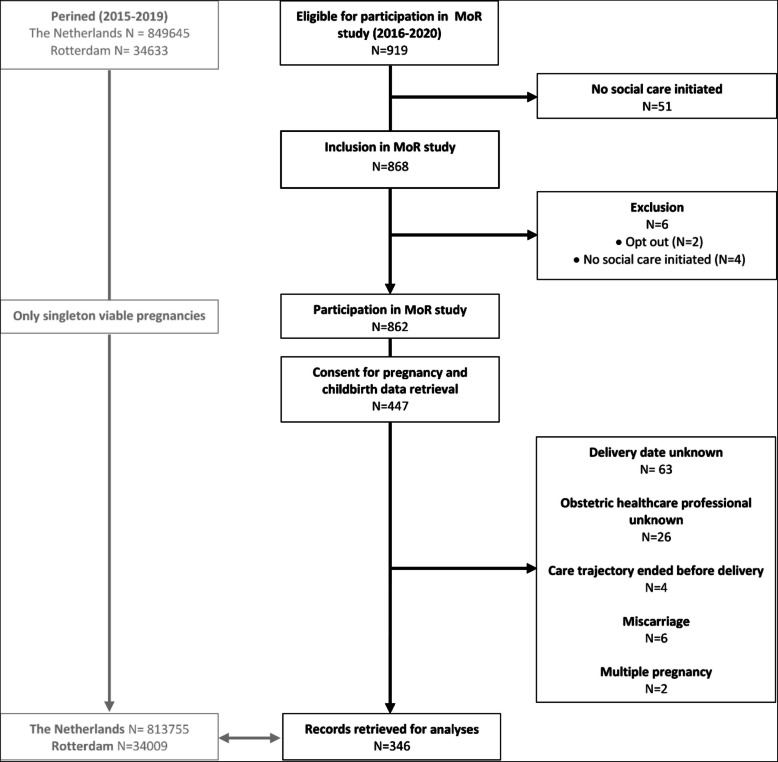
Flowchart study population

Social care professionals assessed eligibility of 919 pregnant women applying for social care between January 2016 and December 2020, of which 862 pregnant women were included in the MoR study (Fig. 1). Written consent for retrieval of pregnancy and childbirth data was given by 447 of the participating women. In 97 of these women data retrieval was not possible due to unknown delivery date or unknown obstetric care provider. Two women had a multiple pregnancy and in six women the pregnancy was not considered viable. Eventually, records of 346 pregnancies and childbirths were used in the analyses.

The baseline maternal (social) and pregnancy characteristics of the MoR population are described in Table 1. Participating women were on average 27.7 (sd 6.1) years of age and 12% of them were aged under 20 years. The median gestational age was 23.3 weeks (IQR 15.9–30.1) at application for social care. The majority of women was nulliparous (54%), resided in a deprived neighbourhood (51%), and had a non-western ethnicity (52%). The marital status was indicated as being single in 21% and as “other” (e.g. unmarried, long distance relationship) in 38%.

**Table 1:** Baseline and social characteristics

	***Mothers of Rotterdam***	***The Netherlands***	***Rotterdam***
*Total number of births in study period*	*346*	*813,755*	*34,009*
*Gestational age at application (median, IQR)*	23.3 (15.9–30.1)	*NA*	*NA*
***Maternal age***			
*Mean (sd)*	27.73 (6.14)	30.78 (4.74)	30.68 (5.20)
< *20*	42 (12.1)	12,539 (1.5)	893 (2.6)
*21–25*	81 (23.4)	94,069 (11.6)	4,857 (14.3)
*26–30*	116 (33.5)	283,619 (34.8)	10,536 (31.0)
*31–35*	66 (19.1)	292,665 (36.0)	11,537 (33.9)
> *35*	41 (11.8)	130,863 (16.1)	6,186 (18.2)
***Parity***			
*Nulliparous*	185 (53.5)	359,462 (44.2)	15,681 (46.1)
*Primi-/Multiparous*	161 (46.5)	454,293 (55.8)	18,328 (53.9)
***Neighbourhood***			
***Non-deprived***	169 (48.8)	75,8415 (93.2)	21,445 (63.1)
***Deprived***	177 (51.2)	54,881 (6.7)	12,564 (36.9)
*Missing*	0 (0.0)	459 (0.1)	0 (0.0)
***Ethnicity***			
***Western***	71 (20.5)	72,2324 (88.8)	25,019 (73.5)
***Non-Western***	179 (51.7)	81,145 (9.9)	8,663 (25.5)
*Unknown*	96 (27.8)	10,286 (1.3)	327 (1.0)
***Marital status***^a^			
*Married*	62 (17.9)	1,652,141 (37.3)	Data not available
*Divorced*	2 (0.6)	348,577 (7.8)	
*Single*	73 (21.0)	-	
*Other*^b^	130 (37.6)	2,426,251 (54.8)	
*Unknown*	79 (22.9)	0 (0.0)	

Data shown as frequency (%), unless otherwise stated

^a^ Data for the Netherlands is derived from Statline (*n* = 4,424,269), here “single” is embedded in the category “Other”

^b^ Including unmarried and long distance relationship

### The Netherlands and Rotterdam

National childbirth outcomes of 813,755 singleton viable pregnancies were available for analyses between January 2015 and December 2019, of which 34,009 were located in Rotterdam (Fig. 1). Compared to the MoR population, women in the Netherlands and Rotterdam were on average older. The majority of pregnant women was multiparous, resided in a non-deprived neighbourhood and had a western ethnicity (Table 1).

### Childbirth characteristics

The majority of women in the MoR population delivered in a hospital (87%; both first and second tier of care), and 2% of the women delivered at home (Table 2). The delivery ended with an emergency caesarean section in 40 women (12%) and with vacuum assistance in 23 women (7%). Postpartum haemorrhage was documented in 11 women (3%).

**Table 2:** Childbirth characteristics

	***Mothers of Rotterdam***	***The Netherlands***	***Rotterdam***
*Total number of births in study period*	*346*	*813,755*	*34,009*
***Location of childbirth***			
*At home*	7 (2.0)	104,955 (12.9)	1,700 (5.0)
*Hospital (first* + *second line care)*	302 (87.3)	681,468 (83.8)	28,961 (85.2)
*Birthing centre*	29 (8.4)	23,117 (2.8)	3,029 (8.9)
*Unknown*	8 (2.3)	4,225 (0.5)	319 (0.9)
***Method of childbirth***			
*Spontaneous vaginal delivery*	240 (69.4)	424,018 (52.1)	15,764 (46.4)
*Vaginal delivery after induction*	13 (3.7)	177,470 (21.8)	8,346 (24.5)
*Scheduled Caesarean Section*	25 (7.2)	64,489 (7.9)	2,711 (8.0)
*Emergency Caesarean Section*	40 (11.6)	63,772 (7.8)	2,999 (8.8)
*Vacuum assisted delivery*	24 (6.9)	59,426 (7.4)	2,734 (8.0)
*Unknown*	4 (1.2)	24,580 (3.0)	1,455 (4.3)
*Post-partum haemorrhage*^a^	11 (3.2)	51,869 (6.5)	1,740 (5.1)
***Perinatal outcomes***			
*Preterm birth (PTB)*	15 (4.3)	50,560 (6.2)	2,172 (6.4)
*Small for gestational age (SGA)*	73 (21.1)	81,639 (10.0)	4,483 (13.2)
*Perinatal mortality*^b^	0 (0.0)	4,242 (0.5)	190 (0.6)
*Low Apgar score*^c^	3 (0.9)	15,810 (1.9)	726 (2.1)
*Congenital anomalies*	20 (5.8)	16,775 (2.1)	673 (2.0)

*Data shown as frequency (%)*

^a^ *defined as blood loss > 1000 ml within the first 24 h following childbirth*

^b^ *defined as mortality between 22 weeks of gestation till 7 days after birth*

^c^ *defined as an Apgar score < 7, 5 min after birth*

In comparison, women in The Netherlands and Rotterdam delivered less often in a hospital (85% and 84%, respectively) and more often at home (13% and 5%, respectively). The number of emergency caesarean sections was lower (8% and 9%, respectively), whereas the percentage of both vacuum assisted deliveries (7% and 8%, respectively) and postpartum haemorrhage (7% and 5%, respectively) were higher compared to the MoR population.

### Crude prevalence rates of perinatal outcomes

**Fig. 2:**
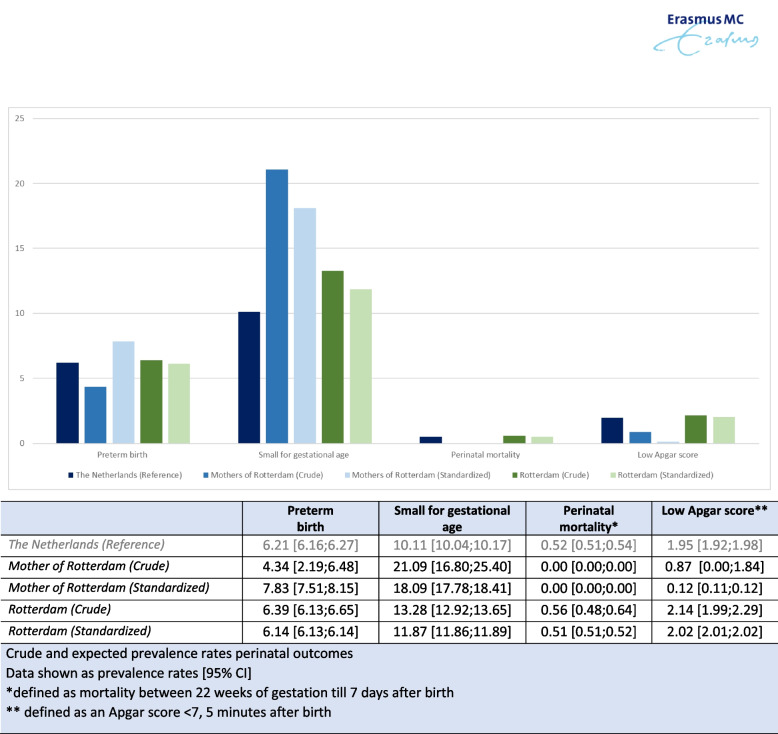
Crude and expected prevalence rates perinatal outcomes

The crude prevalence rates of the studied perinatal outcomes are displayed in Fig. 2. The prevalence of PTB (4.34% (95%CI 2.19–6.48)) was lower in the MoR population compared to the Netherlands (6.21% (95%CI 6.16–6.27)) and Rotterdam (6.39% (95%CI 6.13–6.65)). The prevalence of SGA (21.09% (95% CI 16.80–25.40)) was higher in the MoR population compared to both the Netherlands (10.11% (95%CI 10.04–10.17)) and Rotterdam (13.28% (95%CI 12.92–13.65)).

There were no cases of perinatal mortality registered in the MoR population. The prevalence of a low Apgar score (0.87% (95%CI 0.00–1.84)) was lower in the MoR population.

### Expected prevalence rates perinatal outcomes

Figure 2 shows the expected prevalence rates for PTB and SGA in the MoR population 7.83% (95%CI 7.51–8.15) and 18.09% (95%CI 17.78–18.41), respectively. They are both higher compared to the expected prevalence rates in Rotterdam. Compared to the crude prevalence, the expected prevalence of PTB was higher, and that of SGA was lower. The expected prevalence of a low Apgar score was even lower than the crude prevalence.

## Discussion

Our study found an unexpected low prevalence of preterm birth and high prevalence of SGA in the MoR population compared to the Netherlands and Rotterdam. Especially since the expected prevalence rates were higher for both outcomes compared to the Netherlands and Rotterdam. This discordance between the observed and expected prevalence rates suggests that the faced adversities have an independent influence on the birth outcomes.

There is a vast body of literature indicating higher prevalence rates of PTB, SGA and perinatal mortality in low SES women than in high SES women [19, 20]. These findings are consistent for different ways of defining SES, for example living in disadvantaged neighbourhoods, low income, low educational attainment or belonging to lower occupational or social classes, with odds ratios (ORs) for adverse perinatal outcomes up to 1.41[19, 21]. When comparing the (unadjusted) odds of PTB and SGA between the MoR population and the Netherlands, we observed ORs of 0.68 for PTB and 2.42 for SGA. However, an overlap was seen in the 95% CI of PTB, suggesting that the observed finding might be attributed to random chance.

The exact mechanism through which disadvantaged circumstances or low SES affect perinatal health is yet unknown. A likely mechanism is stress, as disadvantaged circumstances are associated with increased stress levels, and exposure to stress is linked to adverse birth outcomes [22–24]. Despite facing a wide variety of adversities with a complex interplay, the majority of the women in the MoR study rated their stress levels as normal [25]. This finding suggests that the women might have developed (dissociative) coping skills over time, which could explain the discordance between the observed and expected prevalence rates of PTB. Repeated exposure to homotypic stressors can lead to psychological and physiological adaptation, where continuous exposure to certain circumstances can become the new norm (i.e., adaptive preferences and habituation) [26–28]. As a result of these processes, the frequency and intensity of cortisol spikes may decrease, resulting in less PTB than expected.

In addition, the lower prevalence of PTB might have contributed to the higher prevalence of SGA. Children who would have otherwise been born prematurely with a low but normal birthweight now experience prolonged exposure to the suboptimal environment, increasing their susceptibility for low birthweight. However, to test whether this is the case intra-uterine growth data is needed, which was not available in this study.

Regardless of the type of care, we only expect limited effects of social care on perinatal outcomes. Although some benefits (e.g., access to resources) may be observed in some cases, other impacts, such as better health outcomes and a reduced incidence of chronic conditions, often take more time to become evident. Moreover, the median gestational age at application was 23.3 weeks in the MoR population. Given that most embryonic development occurs in the first trimester of pregnancy, social care should start as soon as possible in pregnancy to effectively influence perinatal outcomes [29].

The findings in our study underline the complex pathophysiology of both PTB and SGA. Even though literature indicates higher prevalence of both outcomes in vulnerable populations, our results do not align. The same is seen in more recent studies in refugees and nomads (i.e. vulnerable populations in disadvantaged circumstances) also finding inconclusive results about the prevalence rates of PTB and SGA among women living in war zones/areas of conflict [30–32]. Interestingly, a recent Dutch study in forced migrants, another highly vulnerable population, found similar prevalence rates of PTB and SGA compared to their reference population [33]. This pattern is consistent with what we see in the MoR population.

### Clinical and research implications

This study shows that socioeconomic disadvantaged circumstances not only affect the highly vulnerable pregnant women, but also their (unborn) children with a higher risk of adverse perinatal outcomes. Even though a lower prevalence of PTB was found, which seems more favourable, this does not mean that these children are better off with their health nor that the disadvantaged circumstances protect for PTB or other adverse perinatal outcomes. Moreover, preterm and SGA new-borns have an increased risk of perinatal complications and significant long term health implications [34–36]. Considering this, children of highly vulnerable women are essentially already doubly disadvantaged in life before it even starts.

It is crucial to acquire a comprehensive understanding of the challenges highly vulnerable women face, and the intricate associations involved. This will help us to optimize care provision and tailor interventions effectively. The findings in our study already raise a lot of new questions, which might help us in understanding the mechanisms resulting in adverse perinatal outcomes. Since it is believed that psychological and physiological adaptation to stress plays a role in this highly vulnerable population, measuring this could enable us to further study this association. Data on perinatal outcomes of highly vulnerable women should also contain information on miscarriages, placental health at labour and accuracy of gestational age determination to better understand underlying mechanisms.

### Strengths and limitations

A major strength of the MoR study is the relatively high number of highly vulnerable pregnant women that were included into the study. However, we could not eliminate the risk of selection for this sub-study specifically. Nevertheless, our previous paper showed no substantial differences in the baseline characteristics and adversities between participants with and without consent [25].

By using data from Perined we were able to provide more context to our study findings and to calculate expected prevalence rates, giving insights into the impact of case-mix and the disadvantaged circumstances on the perinatal outcomes. Unfortunately, data on several variables (e.g. miscarriages, placental health, cortisol levels) were not available. Therefore, we were unable to correct for their influence on the perinatal outcomes or calculate adjusted ratios.

While the precise mechanism linking disadvantaged circumstances or low socioeconomic status (SES) to perinatal outcomes is not fully understood, various contributing factors have been identified [8–10]. These encompass environmental, financial, and medical aspects, including pollution, neighbourhood safety, lack of access to healthcare, infections, and placental disorders. Unfortunately, data on these factors were unavailable for the MoR population, and there were numerous missing data in the National registries. Therefore, we could not assess these factors.

Lastly, we were not able to assure mutual exclusivity of the pregnancies. Ideally, we would have identified the MoR pregnancies in the Perined dataset, which would also have enabled us to conduct multivariable regression analyses, however, this was not possible due to insufficient unique identifiers. Since the MoR population would maximum account for 0.04% of the cases in Perined the expected impact was negligible.

## Conclusions

Our study revealed unexpectedly low rates of PTB and high rates of SGA among the MoR population. The exact pathophysiological mechanisms via which prolonged exposure to unfavourable conditions during pregnancy influences perinatal health is more complex than previous literature suggests. More in depth research into these mechanisms is needed to ultimately optimize care for highly vulnerable pregnant women and their children.

## Supplementary Information

Supplementary Material 1.

## Data Availability

The datasets used and/or analyzed during the current study available from the corresponding author on reasonable request.
