# Supplementary material for: Incidence of adverse perinatal outcomes in highly vulnerable pregnant women – the Mothers of Rotterdam study
Source: BMC Pregnancy Childbirth. 2025 Apr 11;25:429. doi: 10.1186/s12884-025-07401-w (PMC11992883; doi:10.1186/s12884-025-07401-w)
Supplement: Supplementary file 1 — Supplementary Material 1. [file 12884_2025_7401_MOESM1_ESM.pdf]

Appendix 1: Vulnerability checklist

Problem domain	Situation	Yes / No
Pregnancy	No prenatal /antenatal care	
	Young and/or uninformed regarding maternity / pregnancy	
	Medical problems in unborn child	
	Negative perceptions of the pregnancy	
	Fear of childbirth	
	No reasonable expectations of maternity	
	Unhealthy lifestyle (e.g. nutrition, hygiene, circadian rhythm)	
Residence	Homelessness (e.g. sleeping outside or in homeless shelter	
	No fixed abode (e.g. spending the night at family or friends)	
	Imminent eviction	
	No gas / water / electricity	
	Overdue maintenance	
	Unsanitary / unsafe residence	
	Illegal situation (e.g. not registered at address)	
Administration and finance	Insufficient income	
	Debt	
Work and education	Unemployed	
	No meaningful daily occupation	
	Illiteracy	
	< 7 years of education	
	Only primary education	
	No secondary or further education	
Parenting	Attachment problems	
	(Expected) parenting problems	
	(Imminent) outplacement of this or earlier children	
	Child Protection Order in place already	
	No basic layette	
Health	Overweight / Underweight	
	Smoking	
	Soft drugs	
	Hard drugs	
	Alcohol	
	Psychiatric problems	
	A lot of stress	
	Intellectually disabled	
	No health insurance	
	Physical problems	
Social functioning	Inadequate social network	
	Single mother	
	Poor communicative skills	
	Language impotent	
	Not self-sufficient (Incapable of finding the right sort of help)	
	Trouble with household chores	
Safety and crime	Detention (history) of the mother	
	Detention (history) of the father	
	Illegal immigration status	
	Domestic violence	
